# Molecular and Serological Findings in Sheep During Two *Coxiella burnetii* Outbreaks in Sicily (Southern Italy)

**DOI:** 10.3390/ani14223321

**Published:** 2024-11-19

**Authors:** Valeria Blanda, Giuseppina Chiarenza, Ilenia Giacchino, Sergio Migliore, Santina Di Bella, Francesco La Russa, Valeria Vaglica, Rosalia D’Agostino, Francesca Arcuri, Carmela Sciacca, Marilena Alfano, Natalia Sciortino, Alessandra Torina, Francesca Grippi, Domenico Vicari

**Affiliations:** 1 Istituto Zooprofilattico Sperimentale della Sicilia, Via Gino Marinuzzi 3, 90129 Palermo, Italy; valeria.blanda@izssicilia.it (V.B.); giuseppina.chiarenza@izssicilia.it (G.C.); francesco.larussa@izssicilia.it (F.L.R.); francesca.grippi@izssicilia.it (F.G.); domenico.vicari@izssicilia.it (D.V.); 2 Azienda Sanitaria Provinciale di Agrigento, Viale della Vittoria, 321, 92100 Agrigento, Italy; 3 Independent Researcher, 90100 Palermo, Italy

**Keywords:** *Coxiella burnetii*, sheep, outbreak, prevention, raw milk, Sicily

## Abstract

Q fever is a widespread zoonotic disease caused by the obligate intracellular bacterium *Coxiella burnetii*, primarily transmitted through the inhalation of contaminated aerosols. This study aimed to detect *C. burnetii* in two Sicilian sheep flocks, with no better defined reproductive disorders reported by the farmers. Blood, individual and bulk milk, ticks, and conjunctival swabs were collected from both flocks (A and B). Real-time and traditional PCRs were carried out to detect *C. burnetii* DNA and anti-*C. burnetii* antibodies were searched using an ELISA. In terms of Farm A, *C. burnetii* DNA was detected in 7.1% of blood samples, 20% of individual milk samples, bulk milk, 66.6% of conjunctival swabs, and in all the examined tick pools. Anti-*C. burnetii* antibodies were found in 77.0% of sera, 92.5% of individual milk samples, and bulk milk. In terms of Farm B, *C. burnetii* DNA was detected in 3.8% of blood samples, 39.4% of individual milk samples, bulk milk, 100% of conjunctival swabs, and in all tick pools; anti-*C. burnetii* antibodies were present in 53.6% of sera, 73.2% of milk samples, and in bulk milk. Our results highlight the high diffusion of *C. burnetii* in the two outbreaks, with widespread pathogen circulation, significant shedding in dairy products, and high environmental contamination, highlighting the need for enhanced surveillance and control measures in dairy sheep farms.

## Simple Summary

Q fever, caused by *Coxiella burnetii*, is a global zoonosis, mainly spread through the inhalation of contaminated aerosols. This study investigated the presence of *C. burnetii* in two Sicilian sheep flocks that were affected by occasional reproductive disorders, as reported by the farmers. Blood, milk, ticks, and conjunctival swabs were sampled from both farms. Real-time and traditional PCRs were used to detect *C. burnetii* DNA, while anti-*C. burnetii* antibodies were analyzed using an ELISA. In both flocks, *C. burnetii* DNA was widely detected in blood (7.1% and 3.8% for Farm A and B, respectively), individual milk (20% and 39.4%), two bulk milk samples, swabs (66.6% and 100%), and in all tick pools. Anti-*C. burnetii* antibodies were found in sera (77.0% and 53.6% for Farm A and B, respectively), individual milk (92.5% and 73.2%), and bulk milk. The study showed widespread pathogen circulation, significant shedding in dairy products, and high environmental contamination. Surveillance and control measures are recommended to mitigate public health risks associated with *C. burnetii* in dairy sheep farms.

## 1. Introduction

*Coxiella burnetii* is an obligate intracellular, small gram-negative bacterium, belonging to the Legionellales order [1]. It is the causative agent of Q fever in humans, in various countries. In domestic ruminants, the disease is called coxiellosis [2] and it has been linked to reproductive disorders, such as late-term abortions, premature births, endometritis, and infertility, making it a major concern for animal health and productivity. Subclinical infection with *C. burnetii* in ruminants is far more common than clinical infection, especially in sheep and goats [3].

While the primary reservoirs of *C. burnetii* include wild and domestic mammals, birds, and arthropods, like ticks, the role of ticks in the transmission of the disease remains the subject of debate [4]. Domestic ruminants are considered the main source of human infections. The introduction of *C. burnetii* into a farm can lead to the spread of infection within the flock or herd, in some cases, resulting in abortion storms. The primary mode of transmission to humans is through the inhalation of aerosols contaminated with *C. burnetii* from infected animals, particularly during parturition, when the pathogen is released in large quantities through placental tissues, milk, feces, and urine [5]. The shedding of *C. burnetii* can persist for multiple weeks following abortion or normal parturition, with a higher bacterial load observed in animals that abort compared to those that give birth normally [6]. The presence of *C. burnetii* in infected animals, along with their movement within indoor facilities, promotes the generation of contaminated aerosols [7]. Bacterial concentrations in aerosols peak during periods of high abortion rates [8] and are also associated with the number of shedding animals in the flock [9]. The degree of dissemination is affected by variables, such as the immune condition of the animals, the size of the flock or herd, and the virulence of the *C. burnetii* strain [10,11]. The natural progression of *C. burnetii* infection across successive breeding seasons in sheep flocks has not been fully elucidated and the duration of infection persistence within a flock remains uncertain [7].

Therapeutic and preventive measures in small ruminants are aimed at reducing abortion rates and bacterial shedding, thereby aiming to reduce environmental contamination [3].

Q fever is an emerging zoonosis and several human Q fever outbreaks have been linked to sheep and goats [12]. Recent evidence from a large outbreak in the Netherlands, with over 3500 notified cases in the Dutch population, indicates that this disease has the potential to become a significant public health concern [13]. In humans, Q fever typically presents as an acute febrile illness with nonspecific symptoms, such as high fever, respiratory infections, eye inflammation, and severe headaches. In some cases, the infection may become chronic, potentially leading to severe complications, like endocarditis and hepatitis. Moreover, in humans, acute and chronic Q fever are frequently misdiagnosed and underreported [14,15,16].

Coxiellosis diagnosis in animals cannot be based on a single diagnostic test, but the clinical and epidemiological context of the case must be examined and considered [17]. In any case, confirmation of the clinical suspicion of coxiellosis requires the application of multiple diagnostic techniques and samples taken from more than one animal. In Italy, most data on the prevalence of coxiellosis in ruminants primarily concern animals experiencing reproductive issues, especially those where abortion is the predominant clinical symptom [18,19,20]

Given the occupational risk for individuals in close contact with infected animals, such as farmers, veterinarians, and laboratory personnel, coxiellosis surveillance and control in livestock are essential. In particular, the identification of shedders is crucial to avoid infections in humans and to prevent diffusion among farmed animals [14].

In this study, we investigate the presence and spread of *C. burnetii* in two Sicilian sheep flocks and dairy products, in a context involving a high level of interconnection between animals, humans, and environmental health.

## 2. Materials and Methods

### 2.1. Farms

**Figure 1:**
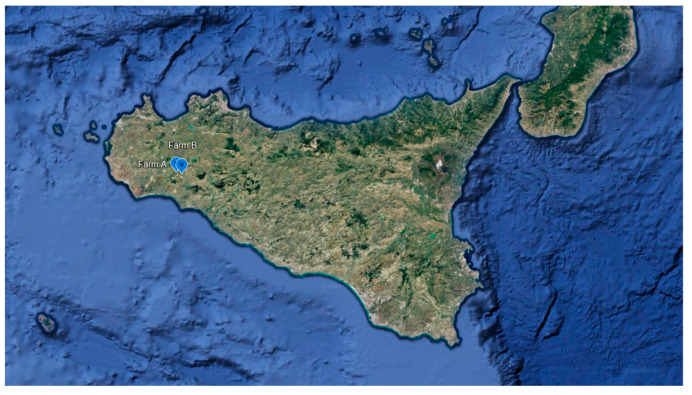
Spatial location of the two sheep farms in Agrigento province (Sicily).

The study concerned two Valle del Belìce dairy sheep flocks, located in Agrigento province (Sicily, Southern Italy) (Figure 1). The two farms are located at a distance of about 4 km from each other and they can be considered a single epidemiological unit, as they are managed by the same family. Valle del Belìce sheep is the most widespread autochthonous Sicilian dairy breed, as well as being the main source of high-quality milk in typical dairy products; notably, Sicilian pecorino is obtained from raw Valle del Belìce milk. The flock’s milk was used on-farm for artisanal cheese manufacturing.

Both farms are located in semi-hilly territory (approximately 200 m above sea level) and are characterized by semi-extensive breeding, characterized by pastures, sometimes shared with other flocks, and a supplementary diet, especially in the dry seasons. The sheep are kept in shelters during the night and for milking and are led out to pasture during the rest of the day.

In both farms, reproduction management was based on the natural reproductive cycle of sheep in the Mediterranean basin, which allows for the concentration of births in the most profitable periods of the year (the production of milk-fed lambs for Christmas and Easter). To obtain births in autumn and stimulate the resumption of the ovarian cycle, rams are re-introduced into the flock after at least 8 weeks of isolation (male effect) during the period of anoestrus in ewes (usually between March and April), while to obtain births in spring, the male is introduced in autumn, exploiting the natural cyclicity of ewes. At the time of sampling (March 2021), most of the ewes on both farms had given birth in late autumn and were lactating, the samples were taken randomly from this breeding group. No anamnestic information on the reproductive disorders of the individual sheep was provided by the breeders.

In neither farm was the health and nutritional status of the flock monitored. In particular, reproductive and feeding techniques were not applied to optimize the results in terms of fertility and prolificacy. Consequently, the reproductive results were not adequately monitored during the breeding season and the breeders reported, in an undefined manner, a decline in fertility and occasional abortions. Both the flocks also showed problems related to tick infestation. At the time of the first inspection, there were 250 and 550 sheep on farms A and B, respectively.

### 2.2. Samples

In March 2021 (T0), an on-farm inspection was carried out on both farms and the samples reported in Table 1 were collected.

Since no epidemiological data were available, the sample size for the serological investigation was calculated considering an expected prevalence of 50%, according to WinEpi software (http://www.winepi.net/, accessed on 1 March 2022) (5% of precision and a 95% confidence level). For both the farms, individual milk samples were collected from all the sheep with a history of hypofertility, as reported by the farmers, and from the same number of healthy sheep.

In particular, samples collected from Farm A included: 126 EDTA-treated and untreated blood samples, 40 individual milk samples, and a bulk milk sample. For Farm B, the collected samples included: 293 EDTA-treated and untreated blood samples, 71 individual milk samples, and a bulk milk sample. Moreover, 15 and 40 tick specimens were collected, respectively, for Farm A and Farm B. Since some sheep showed tearing and discolored wool or hair below the eye caused by serious ocular discharge, 3 and 8 conjunctival swabs were collected from Farm A and B, respectively, from symptomatic animals.

In August 2021 (T1), a second set of samples was conferred to our Institute (Table 1). From Farm A, the collected samples included: 3 blood samples, 2 individual milk samples, and 3 conjunctival swabs. From Farm B, the collected samples included: 6 blood samples, 5 individual milk samples, a bulk milk sample, and 6 conjunctival swabs.

The samples were stored at +4 °C, until processing. The EDTA-untreated blood samples were centrifuged at 1500× *g* for 10 min, at room temperature, for serum separation. The serum samples were frozen at −20 °C, until further serological analyses.

### 2.3. Morphological Identification of Ticks

The collected ticks were kept alive in the laboratory for a week, at room temperature, in order to allow the ectoparasites to cleanse themselves of any ingested blood. The species, sex, and status identification of the ticks followed standard morphological observations [21]. Once morphologically identified, the ticks were stored at −20 °C, until further examination. Each tick was bathed in 70% ethanol for 5 min and divided lengthwise into two parts, in sterile Petri dishes, under a stereomicroscope, using sterile forceps and scalpels: one half was screened using molecular methods, with the remaining half kept in alcohol, pending further investigation. The ticks belonging to the same species and stage, and derived from the same animal, were grouped in pools.

### 2.4. DNA Extraction

The conjunctival swabs were placed in 1 mL of physiological solution for 1 h; DNA extracted from 0.2 mL of the medium was placed in 0.2 mL of Lysis Buffer and 0.02 mL of Proteinase K at 55 °C for 1 h.

Cells from the milk were obtained by centrifugation for 30 min of 10 mL at 3500× *g*; a 0.1 mL pellet was collected and added to 0.18 mL of Digestion Buffer and 0.02 mL of Proteinase K; the samples were stored at 55 °C overnight.

DNA was extracted from 0.2 mL of blood, added to 0.2 mL of Lysis Buffer and 0.02 mL of Proteinase K and stored at 55 °C for 10 min.

The ticks were sectioned longitudinally and one half of each tick was used for DNA extraction, after overnight incubation in 0.18 mL of Genomic Digestion Buffer and 0.02 mL of Proteinase K. The remaining half of each tick was preserved in 70% ethanol. DNA extraction was carried out using a PureLink Genomic DNA Kit (Thermo Fisher Scientific, Carlsbad, CA, USA), according to the manufacturer’s instructions.

### 2.5. Molecular Investigations

Real-time PCRs were carried out on the DNA extracted from the collected samples to detect DNA from *C. burnetii* [22], *Chlamydia* spp. [23], *Neospora caninum* [24], and *Toxoplasma gondii* [25]. For the *C. burnetii* real-time PCR, samples were considered positive when they showed cycle threshold (Ct) values of 38 or lower. Positive results were also confirmed by a conventional PCR [26]. Real-time PCRs were carried out using a Bio-Rad CFX96 System (Bio-Rad Laboratories, Hercules, CA, USA) and a Quantum Studio 6 Flex, Applied Biosystem (Thermo Fisher Scientific, Carlsbad, CA, USA). Traditional PCRs were carried out using a SimpliAmp Thermal Cycler Applied Biosystem Kit (Thermo Fisher Scientific, Carlsbad, CA, USA).

### 2.6. Microbiological Analysis of Swabs and Milk

In addition to molecular analyses, the swabs were processed using bacteriological techniques to isolate the bacterial species. Cultures were performed using selective and differential media, including blood agar, MacConkey agar, and mannitol salt agar (Thermo Fisher Scientific, Waltham, MA, USA), to support the growth of both commensal and pathogenic species present in the collected samples [27,28]. Colonies isolated from these media were subsequently identified through biochemical and enzymatic assays.

### 2.7. Serological Analyses

Antibodies against *C. burnetii* were searched using an ID Screen^®^ Q Fever Indirect Multi-species ELISA (Innovative Diagnostics SAS, Grabels, France) in regard to the serum, individual and bulk milk samples, according to the manufacturer’s instructions.

The optical density (OD) of the tested samples and controls was measured using a Multiscan Labsystem spectrophotometer (model Ex) at 460 nm. For each sample the S/P % percentage was calculated using the following formula:

S/P % = (OD sample − OD nc)/(OD pc − OD nc) × 100.

Regarding brucellosis, the detection of anti-Brucella spp. antibodies in the serum of the sampled animals was performed using the Rose Bengal Test (RBT), as it is the serological method listed among the official methods and recommended in the WOAH Manual [29].

### 2.8. Statistical Analysis

Statistical analysis was performed using the Chi-square test (2 × 2 contingency table). The serological and molecular prevalences in the milk and blood samples were compared among the two farms, with a significance level of *p* ≤ 0.05.

### 2.9. Ethical Statement

All the tests were carried out in accordance with the relevant guidelines and regulations. Biological samples were taken from animals that were suspected of infection below the threshold of the directive. For the purpose of this study, permissions from the farmers were sought in advance for the use of the samples and for the collection of ticks from the sheep affected by tick infestation.

## 3. Results

Both the molecular and serological analyses carried out on the blood samples showed a high prevalence for *C. burnetii*, as detailed in Table 2 and Table 3. In particular, *C. burnetii* DNA was detected in 9/126 (7.1%) and 11/293 (3.8%) of EDTA blood samples from Farm A (Ct value 36.5 ± 1.4) and B (Ct value 36.2 ± 1.8), respectively. Concerning individual milk samples, *C. burnetii* DNA was detected in 8/40 (20%) samples from Farm A (Ct value 36.0 ± 1.4) and in 28/71 (39.4%) samples from Farm B (Ct value 35.0 ± 1.7). In both the farms, the bulk milk samples showed positive results for *C. burnetii* DNA (Ct values of 33.7 and 31.0 for Farm A and B, respectively).

All the collected ticks were identified as *Rhipicephalus sanguineus* s.l. and grouped into 12 pools (four for Farm A and eight for Farm B), according to sex, collection site, and host. All the analyzed pools showed positive results for *C. burnetii* DNA (Ct values of 36.3 ± 1.7 and 36.4 ± 1.6 for Farm A and B, respectively).

Interestingly, 2/3 (66,6%) and 8/8 (100%) of the swabs, respectively, from Farm A (Ct value 35.7 ± 1.6) and B (Ct value 36.1 ± 1.8), were positive for *C. burnetii* DNA.

Additionally, we carried out serological investigations to detect anti-*C. burnetii* antibodies in the serum and milk samples. Antibodies against *C. burnetii* were detected in 97/126 (77.0%) and 157/293 (53.6%) sera from Farm A and B, respectively. Serological investigations carried out on the milk samples revealed antibodies against *C. burnetii* in 37/40 (92.5%) and 52/71 (73.2%) of individual milk samples collected from Farm A and B, respectively. The bulk milk samples from both farms tested positive for *C. burnetii* antibodies.

Statistical analyses carried out to assess the serological and molecular prevalence in the milk and blood samples between the two farms showed a significant difference (*p* ≤ 0.05) in terms of the serological prevalence in the blood samples (<0.00001) and a molecular and serological prevalence in the individual milk samples (*p*-value 0.036 and 0.015, respectively).

### 3.1. Differential Diagnosis Results

Differential analyses were carried out on the samples collected at T0. In particular, molecular tests carried out on all the blood samples showed negative results for the presence of *Chlamydia* spp., *T. gondii*, and *N. caninum*. The Rose Bengal Test for the detection of anti-*Brucella* antibodies carried out on all sera samples was also negative. Cultural analysis of all the individual and bulk milk samples excluded the presence *Salmonella* spp., *Listeria* spp., and *Campylobacter* spp. Cultural analyses carried out on the conjunctival swabs from the symptomatic sheep detected *Staphylococcus aureus* in 2/3 (66.6%) and 1/8 (12.5%) of the swab samples for Farm A and B, respectively, while *Clostridium* spp., *Pseudomonas* spp., and *Streptococcus* spp. were detected each in 1/8 (12.5%) swabs from Farm B.

### 3.2. Outbreak Management and Legislative Implications

Both farmers involved, due to the high costs, decided not to vaccinate the affected animals. Furthermore, taking into account the almost complete absence of evident clinical symptoms, the farm veterinarian advised against supportive antibiotic therapy.

At the time of the outbreaks, the health measures provided by Italian legislation (Presidential Decree n. 320/1954) required the reporting of the disease as a zoonosis (art. 5) and the implementation of hygiene–sanitary and biosecurity protocols to reduce the spread of infection inside and outside the farm (art. 9).

In addition, regarding outbreak management, specific restrictive measures on farmed animals and milk products have to be adopted only when human Q fever cases relate to exposure to infected animals (art. 142). Fortunately, human cases connected to the affected farms have been excluded as a possibility and the veterinary policing measures have not been applied to the farms.

In late summer (August 2021), a second set of samples was conferred to our Institute. The samples were analyzed using molecular and serological tests for *C. burnetii* (Table 2 and Table 3).

For Farm A, the molecular analyses carried out on the blood and individual milk samples showed negative results, while it was not possible to test the bulk milk sample. In contrast, molecular positivity was still detected in the bulk milk sample (Ct value of 36.6) and in 1/6 (16,7%) of the conjunctival swabs (Ct value of 36.5) from Farm B.

Antibodies were still detected, for Farm A and B, respectively, in 1/3 (33.3%) and 3/6 (50%) of the blood samples, 2/2 (100%) and 5/5 (100%) of the individual milk samples, and in the bulk milk sample from Farm B.

## 4. Discussion

In this study, the presence of *C. burnetii* infection was reported in two sheep farms in Sicily, showing the widespread circulation of the pathogen in both farms.

*Coxiella burnetii* DNA was detected in blood, individual and bulk milk samples, conjunctival swabs, and ticks. In addition, *C. burnetii* antibodies were found in sera and milk samples. A decrease in molecular positivity was registered following the treatment measures undertaken, while the presence of antibodies still remained high, as expected. However, the sample size at T1 was too small to draw any conclusions on the course of infection.

Other studies describe the prevalence of *C. burnetii* in sheep or goats, however the results are often difficult to compare, since they are likely to have been obtained based on different bodily fluids and tissues, methodologies, and study population composition [3]. Studies carried out in Southern Italy, Puglia and Basilicata regions, reported *C. burnetii* as a significant agent for sheep abortions in Italy [20]. A survey carried out in Abruzzo and Molise regions (central Italy) in order to identify the causes of abortions and monitor suspect or positive flocks, also detected a high positivity rate in 2023, with 62 out of 99 goats (62.6%) and 21 out of 24 sheep (87.5%) testing positive [30]. The high positivity rate in this last study is probably due to the high rate of focalized sampling from suspected or confirmed *C. burnetii*-infected flocks, with a history of positive cases. Another study carried out in central Italy, involving 2783 healthy sheep (94 flocks) from an extensive grazing system, reported a seroprevalence of 37.8% and 87.2% at the animal and flock level, respectively [14]. Similar results were obtained in Sardinia in 2018, with an overall prevalence of 34% in small ruminants [31].

In the current study, both Farm A and B showed a high seroprevalence for *C. burnetii* antibodies (77.0% and 53.6%, respectively). Moreover, a significant number of blood samples testing positive for *C. burnetii* (7.1% and 3.8% for Farm A and B, respectively) were obtained by a real-time PCR. This finding is noteworthy, given that *C. burnetii* bacteremia is typically brief. Within days, the bacterium is localized to tissues, such as the mammary glands, uterus, and placenta. Once established, *C. burnetii* is shed into the environment over an extended period through milk, feces, and vaginal secretions [32].

Our results showed that individual milk tested positive for *C. burnetii* DNA, with prevalence values of 20% and 39.4% for Farm A and B, respectively. Moreover, a high prevalence of *C. burnetii* antibodies was detected in milk samples (92.5% and 73.2% for Farm A and B, respectively). Bulk tank milk is a relatively novel sample type for *C. burnetii* investigations in sheep and goats and it can also be applied to ELISA testing to measure immunological responses at the flock level [33]. This non-invasive method proves useful in monitoring herd health [34]. Infected sheep shed *C. burnetii* into milk for variable periods. Their milk can become contaminated with this bacterium, even through fecal matter, vaginal mucus, or urine. However, in our study, the high number of animals with bacteremia more strongly suggests the release of the pathogen into the milk by infected animals, rather than environmental contamination. The growing preference for raw milk products presents a public health concern due to the associated increased risk of milk-borne diseases. The ingestion of contaminated milk or dairy products can be a source of infection in humans, even if the risk of transmission of *C. burnetii* via milk is lower compared to the inhalation of aerosols from birth products or contact with livestock [35]. In particular, pregnant women, children, the elderly, and immunocompromised individuals should avoid contact with unpasteurized products.

Pasteurization effectively inactivates *C. burnetii* in milk, underscoring the importance of this process for preventing milk-borne infections. Although the oral route of transmission should not be overlooked, especially for farmers producing artisanal cheese, pasteurization remains crucial for consumer safety. In this case, the production of cheeses from raw milk produced by both farms involved in this study deserves particular attention in order to limit the risk to the consumer.

The high percentage of positive ticks detected in this study does not, in itself, provide sufficient evidence to support their role as active vectors in the transmission of the pathogen in question. Instead, this result could indicate a high level of environmental contamination by the pathogen in the two farms under investigation. Such contamination may reflect an environment conducive to the spread of this microorganism, as evidenced by its detection in the ticks. However, the possibility that the observed positivity is related to the presence of undigested blood meal residues cannot be entirely excluded, despite the methodological precautions taken to ensure that the ticks had completed digestion prior to the analysis. Notoriously, ticks represent a secondary route of infection in terms of *C. burnetii* in ruminants and vector competence has been demonstrated for many hard and soft tick species, even if the vector capacity is low in field conditions [14]. Surprisingly, we found high positive rates (66.6% and 100% for Farm A and B, respectively) in the conjunctival swab samples collected from animals showing clinical symptoms of ocular discharge. Ocular manifestations of this disease are uncommon and have been rarely documented. Cases of chorioretinitis, uveitis, and bilateral optic neuritis associated with *C. burnetii* infection have been reported in humans [36,37].

The detection of the pathogen’s DNA in sheep conjunctival secretions opens the door to speculations about the potential association of *C. burnetii* and ocular manifestations, even in animals. Although this is an intriguing hypothesis, the current data are insufficient to confirm this possibility. The presence of positive conjunctival swabs for the pathogen’s DNA does not definitively demonstrate a direct etiological relationship between the pathogen and the symptoms observed in some subjects. The identification of co-infections with other bacterial agents commonly associated with the symptoms complicates the attribution of an exclusive pathogenic role to *C. burnetii* in this context. The positive conjunctival swabs may even reflect the high level of environmental contamination observed in the studied farms. Further studies are necessary to determine whether such a route of pathogen elimination through ocular secretions could be epidemiologically significant and whether it plays a role in the transmission of the infection among animals and potentially to humans.

Our results showed a high diffusion of the pathogen within the two outbreaks studied, even if not correlated to the clinical symptoms that appeared to be of mild severity and minimally widespread within the flock. This highlights the importance of diagnostics in regard to subclinical infections.

*C. burnetii* is a multi-host pathogen that is predominantly asymptomatic in animals, though it can lead to reproductive losses in herds [38,39]. As a zoonotic pathogen, identifying the clinical signs of coxiellosis is critical to minimize unnecessary exposure to infectious materials. It has been widely reported that in pregnant animals, the primary clinical manifestations are abortions and stillbirths, while in non-pregnant animals, the infection is generally asymptomatic, making detection difficult. Our data are not sufficient to correlate the reproductive disorders reported by farmers to *C. burnetii* infection. Firstly, due to the lack of anamnestic data regarding the reproductive performance of the flocks before and after infection; secondly, due to the complete lack of rationalized reproductive management (male/female ratio in the flock, health and nutritional control status, recovery of body condition score during lactation, ultrasound control, etc.) and, thirdly, due to the lack of provision of aborted fetuses and fetal envelopes for determination of the cause of abortion.

To date, in the European Union (EU), Q fever is enlisted within the ‘e’ category in Commission Implementing Regulation (EU) 2018/1882 and specific surveillance and rules on notification and reporting are applicable, as defined by Regulation (EU) 2016/429, which entered into force on 21 April 2021. On the other hand, Q fever is a mandatory notifiable disease in humans and all cases are reported through the European Surveillance System [14].

In humans, *C. burnetii* infection may be asymptomatic or manifest as clinical symptoms, such as high fever, eye infections, respiratory tract infections, and severe headaches [16]. In 2019, 23 countries reported 1069 cases, with 90% classified as confirmed, and the highest numbers being from Spain, France, and Germany. Humans primarily acquire the infection through the inhalation of contaminated aerosols or direct exposure to urine, feces, placenta, sperm, and vaginal secretions from infected animals. The infection can also be contracted through the consumption of contaminated raw milk [40], although the link between unpasteurized milk consumption and human disease, as reported above, remains unclear [41].

Given the subclinical and non-pathognomonic clinical manifestation of the disease, coxiellosis in sheep and goats in Italy appears to be underestimated. For this reason, it is of great importance to always consider *C. burnetii* during the differential diagnosis process in the case of reproductive disorders in small ruminant farms.

Determining the antibiotic susceptibility of *C. burnetii* is challenging due to its obligate intracellular nature. In cases of suspected *C. burnetii*-induced abortions in small ruminants, two injections of oxytetracycline during the final month of pregnancy are recommended, though this treatment has shown variable effects on bacterial shedding. Vaccination is the most effective strategy for preventing abortions and reducing bacterial shedding [42]. Vaccines containing phase I *C. burnetii* are particularly effective in reducing shedding in vaginal fluids, feces, and milk, especially when administered before the first pregnancy. However, in naturally infected animals, vaccination may not fully prevent abortion or shedding due to persistent infections [43,44,45].

Good hygiene practices on farms, particularly those involving sheep and goats, are essential for preventing coxiellosis transmission. Airborne transmission plays a significant role in some outbreaks [42]. General farm hygiene remains crucial for reducing human exposure to *C. burnetii*. Since shedding during parturition is the main source of transmission, strict hygiene measures should be implemented during lambing and kidding on infected farms. The proper disposal of placentas and fetuses, composting of manure, and the use of lime or calcium cyanide for manure treatment are recommended to minimize environmental contamination. Further reduction of environmental contamination can be achieved through adopting appropriate tick and helminth control measures [38].

## 5. Conclusions

This study reports on the spread of *C. burnetii* infection in two sheep farms in Sicily, highlighting a scenario involving widespread environmental contamination, even in the absence of significant clinical symptoms. Furthermore, the high presence of the pathogen in milk used for the production of raw milk cheese raises concerns about food safety, although the consumption of milk and dairy products is not considered the primary transmission route. Further targeted investigations are necessary to better understand the epidemiological dynamics of *C. burnetii* infection in small ruminants. Surveillance and control measures in livestock should be prioritized to prevent further public health risks.

## Figures and Tables

**Table 1:** Samples collected from the two investigated farms.

	T0 (March 2021)					T1 (August 2021)			
Farm	Blood Sample	Milk Samples	Bulk Milk	Ticks	Conjunctival Swabs	Blood Sample	Milk Samples	Bulk Milk	Conjunctival Swabs
A	126	40	1	15	3	3	2	-	3
B	293	71	1	40	8	6	5	1	6
Total	419	111	2	55	11	9	7	1	9

**Table 2:** Results of molecular analyses for *C. burnetii* carried out on collected samples at T0 and T1.

	Real-Time PCR *C. burnetii* Positive/Total (%)							
	T0					T1		
Farm	Blood Sample	Individual Milk	Bulk Milk	Tick Pools	Conjunctival Swabs	Individual Milk	Bulk Milk	Conjunctival Swabs
A	9/126 (7.1%)	8/40 (20%)	+	4/4 (100%)	2/3 (66.6%)	0/2 (0%)	n.a.	0/3 (0%)
B	11/293 (3.8%)	28/71 (39.4%)	+	8/8 (100%)	8/8 (100%)	0/5 (0%)	+	1/6 (16.7%)

+: positive.

**Table 3:** Results of serological analyses for *C. burnetii* carried out on collected samples at T0 and T1.

ELISA *C. burnetii* Positive/Total (%)						
	T0			T1		
Farm	Blood Sample	Individual Milk	Bulk Milk	Blood Samples	Individual Milk	Bulk Milk
A	97/126 (77.0%)	37/40 (92.5%)	+	1/3 (33.3%)	2/2 (100%)	n.a.
B	157/293 (53.6%)	52/71 (73.2%)	+	3/6 (50%)	5/5 (100%)	+

+: positive.

## Data Availability

The data are contained within the article.
